# Associations between Bruxism, Stress, and Manifestations of Temporomandibular Disorder in Young Students

**DOI:** 10.3390/ijerph19095415

**Published:** 2022-04-29

**Authors:** Diana Vlăduțu, Sanda Mihaela Popescu, Răzvan Mercuț, Mihaela Ionescu, Monica Scrieciu, Adina Dorina Glodeanu, Andreea Stănuși, Ana Maria Rîcă, Veronica Mercuț

**Affiliations:** 1 Department of Prosthetic Dentistry, University of Medicine and Pharmacy of Craiova, 200349 Craiova, Romania; dianavladutu04@gmail.com (D.V.); andreeacazan22@gmail.com (A.S.); veronica.mercut@umfcv.ro (V.M.); 2 Department of Oral Rehabilitation, University of Medicine and Pharmacy of Craiova, 200349 Craiova, Romania; sanda.popescu@umfcv.ro; 3 Department of Plastic and Reconstructive Surgery, University of Medicine and Pharmacy of Craiova, 200349 Craiova, Romania; razvan.mercut@umfcv.ro; 4 Department of Medical Informatics and Biostatistics, University of Medicine and Pharmacy of Craiova, 200349 Craiova, Romania; 5 Department of Internal Medicine, University of Medicine and Pharmacy of Craiova, 200349 Craiova, Romania; adina.glodeanu@umfcv.ro; 6 Department of Odontology, University of Medicine and Pharmacy of Craiova, 200349 Craiova, Romania; ana.rica@umfcv.ro

**Keywords:** bruxism, stress, anxiety, muscular contractions

## Abstract

Bruxism is a repetitive activity of the masticatory muscles, which determine teeth grinding or clenching, associated with rigidity, bracing, or thrusting of the mandibula. The aim of this study was to determine the prevalence of possible bruxism in 328 students attending the Faculty of Dental Medicine, University of Medicine and Pharmacy of Craiova, and its associations with stress and other manifestations of the temporo-mandibular disorder. This was a questionnaire-based study to collect information on self-evaluation of bruxism presence, frequency of specific episodes, stress, anxiety, and other manifestations of temporo-mandibular disorder. Self-evaluated bruxism was identified in 39.33% from the entire study group, allowing us to define two subgroups for further analysis. Sleep bruxism was present in 16.28% of participants; awake bruxism was present in 68.99%, while 14.73% of participants presented a combined form. The main manifestation of bruxism was reported as teeth grinding. Fatigue was identified as a common clinical sign of bruxism and temporo-mandibular disorder. Group distribution analysis (Chi-Square) indicated significant associations between bruxism and stress, panic, restlessness, or increased stress during the COVID-19 pandemic (*p* < 0.05). Bruxism, and especially awake bruxism, has increased in prevalence among young students, and it has been associated with increased levels of stress.

## 1. Introduction

Some of the most important stresses on the masticatory system (MS) occur during episodes of bruxism. Although it has been known since ancient times [1] and has been extensively studied, bruxism is still a topic of great interest to specialists in various medical fields. For a long time, scientists considered that the etiology of bruxism was mainly based on occlusal factors, as the occlusal interferences represented the local triggers that generated various types of manifestations [2,3]. Later, through experimentally generated interferences, Rugh demonstrated that occlusal corrections did not modify the parafunctional bruxism episodes [4]. Other factors have also been considered: genetics [4], alcohol or tobacco consumption, drugs, or specific medications [5], MS disorder [6], systemic factors (neurological, mental, and even neurotransmitter disorders) [7,8,9], and psychosocial factors (stress, anxiety) [10,11,12], but no clear implications have been found. Therefore, the role of main factors was left to the newly investigated psychosocial and behavioral factors, such as levels of stress and type of personality. In fact, new hypotheses reversed the process, identifying bruxism as involved in the mitigation of stress-induced psychosomatic disorders, and even calling it a “psychic stress valve” [13].

Over time, bruxism has also been considered a manifestation of temporomandibular disorder [1,14,15,16,17], a sleep disorder [7,18], a behavioral disorder, more commonly associated with stress [10,19,20] or even a movement disorder [21].

The consequences of bruxism involve dental wear [2], cracked teeth, hypertrophy of masticatory muscles, exostoses, prosthetic dental failures, all added to a general fatigue state or extreme tiredness and nervosity [22].

In 2012, a group of experts in the field established an international consensus according to which bruxism is defined as a repetitive activity of the masticatory muscles that causes clenching or grinding of the teeth, associated with bracing or thrusting (projecting forward or sideways) of the mandible [23]. On this occasion, classifications of bruxism according to the circadian rhythm were also defined: sleep bruxism (SB) and awake bruxism (AB). A new meeting was held in 2017 to clarify some issues related to the previous definition, to develop separate definitions for the two forms of bruxism, and to determine whether bruxism can be considered a condition or a risk factor [24]. According to this consensus, bruxism should be approached as a contraction disorder of the masticatory muscles with various causes. Regarding these causes, frequently after the consensus from 2012, bruxism was associated with stress [25,26].

This study sought to establish the prevalence of possible bruxism in students attending the Faculty of Dental Medicine, University of Medicine and Pharmacy of Craiova, to identify associations of bruxism with stress and anxiety, as well as to highlight some manifestations of bruxism associated with the temporo-mandibular disorder.

## 2. Materials and Methods

For this study, a questionnaire was completed with questions related to epidemiological data, the presence of possible bruxism episodes, their association with stress, anxiety, sleep disorders (insomnia) and manifestations of the temporomandibular disorder. The questionnaire also included a question regarding the level of stress in the context of the COVID-19 pandemic.

Among the clinical signs that accompany the presence of possible bruxism, the following signs for SB were recorded: grinding of teeth (perceived by partner or personally), fatigue, muscle pain or tenderness, sore throat, headache, insomnia associated with clenching teeth, and for AB: daytime muscular fatigue associated with the functions of the MS, clenching, or grinding of teeth during the day.

Stress was analyzed in the light of the following signs: nervosity, tremor, strong heartbeats, tension, dissatisfaction with current occupation, difficulty initiating sleep (DIS), disturbed sleep (difficulty maintaining sleep) (DS), waking up in the morning very early (EMA—waking up before the desired time, despite the time, and difficulty in falling asleep again), restless sleep (for more than 1 month), feeling tired (at least 3 days a week), sleep deprivation (the need to sleep for more than 1 h compared to normal sleep duration).

Anxiety was analyzed in terms of the following signs: panic, the feeling that something bad is going to happen, feeling scared for no reason, sensation of fear.

Data regarding the presence of clinical signs and assessment of perceived stress were collected through a set of questions with the following possible answers: Yes/No/I don’t know, as well as: Never/Rarely/Sometimes/Often/Continuously, or None/Slightly/Often/Very much/Extensively.

The prevalence of possible bruxism in the studied group was assessed based on self-reporting, and the frequency of bruxism episodes fell under the following categories: sometimes, often, and continuously.

### Statistical Analysis

The validation procedure for our questionnaire included the check for normality distribution and internal consistency verification based on Cornbach’s alpha coefficient. Sampling adequacy was assessed based on the Kaiser–Meyer–Olkin (KMO) measure and Bartlett’s test of sphericity.

The data collected after completing the questionnaires were initially processed using Microsoft Excel (San Francisco, CA, USA). Statistical tests were applied using Statistical Package for Social Sciences (SPSS), version 20 (IBM Corp., Armonk, NY, USA), and included: Chi Square and Cochran–Armitage test of trend for associations between dichotomous and ordinal variables, Mann–Whitney U test of two proportions and Kruskal–Wallis H for group distribution of ordinal and nominal data. The value *p* < 0.05 was considered statistically significant.

The study was conducted after obtaining informed consent from all participants regarding the objectives and conduct of the study, in compliance with the Helsinki Declaration. The approval of the Ethics Commission of University of Medicine and Pharmacy of Craiova was previously obtained (no. 84/03.06.2021).

## 3. Results

The study was conducted in 2021 on 328 students, aged between 21 and 41 years old, of both sexes, attending the Faculty of Dental Medicine, University of Medicine and Pharmacy of Craiova. All participants had a very good health status (no medication or systemic conditions were recorded). The distribution by gender was the following: 212 females (64.63%), and 116 males (35.36%). The age analysis revealed three distinct groups: 139 participants (representing 42.38% from the entire study group) were at most 23 years old, 131 participants (representing 39.94%) were 24 or 25 years old, and 58 participants (representing 17.68%) were aged older than 26 years.

The questionnaire reliability analysis revealed that the value of the overall Kaiser–Meyer–Olkin (KMO) measure was 0.915, classified as “marvelous” within the Kaiser classification of measure values (1974). This indicated that the items were characterized by linear relationships. Bartlett’s test of sphericity was statistically significant (*p* < 0.0005), indicating that the data were likely factorizable. The overall questionnaire had a high level of internal consistency, as determined by a Cronbach’s alpha of 0.915.

### 3.1. Self-Evaluated Bruxism

Regarding the prevalence of bruxism, 129 participants (80 females, 49 males), representing 39.33% of the entire study group, reported the presence of bruxism signs. There was a very weak association between gender and self-evaluated bruxism (φ = 0.044), which was not statistically significant χ^2^(1) = 0.638, *p* = 0.424. A Cochran–Armitage test of trend was run to determine whether a linear trend existed between the age groups and the proportion of participants who reported the presence of bruxism. The proportion of participants with self-evaluated bruxism varied, according to increasing age, from 0.381, to 0.359, ending with 0.500 for those older than 26 years old. The Cochran–Armitage test of trend did not show a statistically significant linear trend between age groups and the proportion of participants with self-evaluated bruxism, *p* = 0.227.

The frequency of bruxism episodes was evaluated as “sometimes”, “often” and “continuously”. The distribution of episode frequency was similar for female and male participants, and there were no statistically significant differences between these groups (U = 1834, z = −0.690, *p* = 0.491). Additionally, the distribution of bruxism episode frequency was similar for the three age groups, and there were no statistically significant differences between them (H(2) = 0.735, *p* = 0.692).

### 3.2. Evaluation of Awake Bruxism, Sleep Bruxism and Combined Bruxism

From the 129 participants with self-evaluated bruxism, 21 participants declared only signs of SB (9 females, 12 males), 89 participants declared only signs of AB (55 females, 34 males), and 19 participants presented both SB and AB (combined bruxism: 16 females, 3 males).

**Figure 1:**
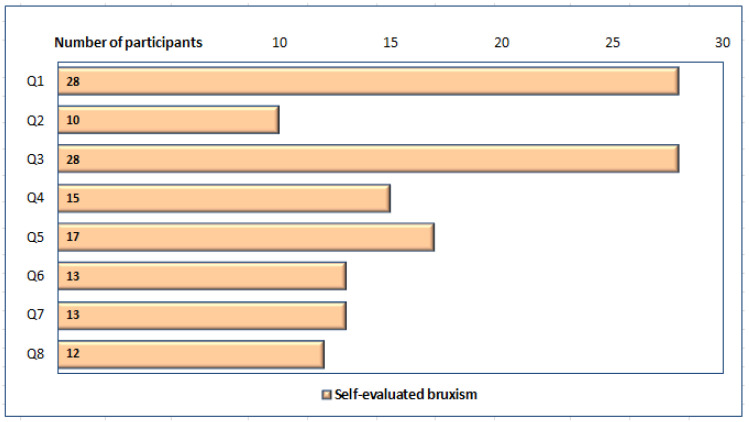
Distribution of participants presenting clinical signs of SB: teeth grinding (reported by the partner of the participant [Q1] or personally [Q2]), muscular fatigue during the day, associated with the functions of the MS [Q3], pain [Q4] or dental sensitivity [Q7], pain of neck muscles in the morning [Q6], headache [Q8], and insomnia related to teeth clenching [Q5].

Overall, for the 40 participants with SB (both the unique and combined forms), the main clinical signs were teeth grinding reported by the partner of the participant [Q1], and fatigue of masticatory muscles sensed in the morning [Q3] (Figure 1).

**Figure 2:**
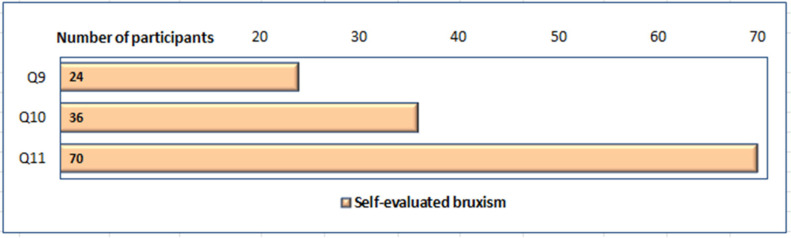
Distribution of participants groups presenting clinical signs of AB: muscular fatigue during the day, associated with the functions of the MS [Q9], grinding [Q10] and clenching [Q11] during daytime.

Overall, of the 108 participants with AB (both the unique and combined forms), 36 reported teeth grinding, 70 reported clenching, and 2 could not clearly report their clinical signs but presented muscular fatigue during the day, associated with the functions of the MS (Figure 2).

The proportion of female participants presenting AB or combined bruxism was higher than the proportion of male participants with the same type of bruxism, and the differences were statistically significant; on the other hand, proportions were similar for SB (Table 1). The distribution of age groups and bruxism episode frequency was similar for participants with or without all three types of self-evaluated bruxism, and there were also no significant differences between these groups (Table 1).

### 3.3. Stress Presence and Associations with Self-Evaluated Bruxism

The evaluation of stress presence (based on responses “often”, “very much”, and “extensively”) revealed that 224 participants (representing 68.29% of the entire study group) reported that they felt stressed. From these 224 participants, 111 (representing 49.55% of participants with self-reported stress; 77 females, 34 males) also presented self-evaluated bruxism, and 113 (50.45%, 75 females, 38 males) did not present self-evaluated bruxism. In summary, 88.80% of participants with self-evaluated bruxism also felt stressed, compared to only 56.78% of participants without self-evaluated bruxism.

**Figure 3:**
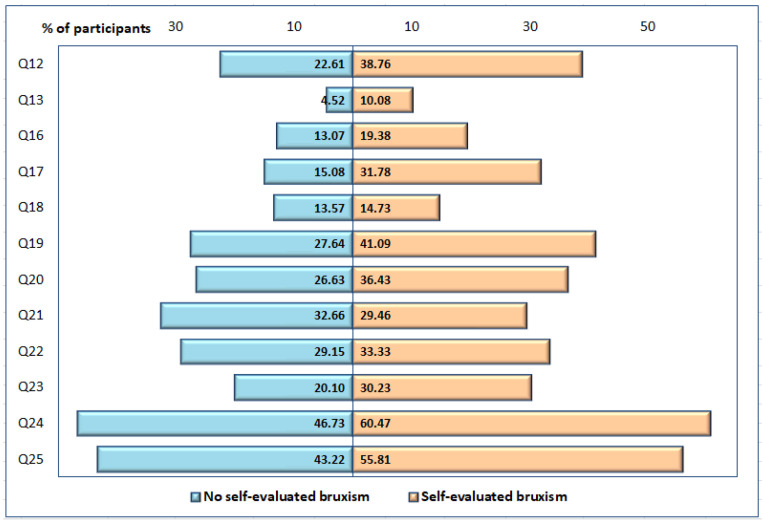
Distribution of participants (expressed as percentage) presenting clinical signs of stress: nervousness [Q12], tremor [Q13], strong heartbeats [Q16], tension [Q17], lack of satisfaction related to current occupation [Q18], DIS [Q19], DS (difficulties maintaining sleep) [Q20], waking up in the morning very early [Q21], EMA [Q22], restless sleep (for more than a month) [Q23], feeling tired (at least three days a week) [Q24], sleep deprivation (the need to sleep for more than one hour beyond current sleep duration) [Q25].

Figure 3 emphasizes the main signs of stress. Overall, stress is especially perceived as a sensation of nervousness and tension.

There was a moderate association between the presence of self-evaluated bruxism and presence of stress (φ = 0.307), which was also statistically significant, χ^2^(1) = 30.950, *p* < 0.0005. On the other hand, there were no statistically significant correlations between stress presence and participant gender or age group (*p* > 0.05).

There were statistically significant differences between the frequency of bruxism episodes and the level of perceived stress, *p* < 0.0005. (Table 2). Subsequent pairwise comparisons were performed based on Dunn’s procedure with a Bonferroni correction for multiple comparisons. This post hoc analysis revealed significant differences in the frequency of bruxism episodes (*p* < 0.0005) for participants who declared the perceived stress level as “none” (predominant frequency “never”), compared to those who declared the perceived stress level as “slightly” (predominant frequencies “never” and “rarely”), “very much” and “extensively” (both with predominant frequencies “sometimes” and “often”).

There were also statistically significant differences between the presence of clinical signs of AB and the level of perceived stress, but not between the presence of clinical signs of SB and the level of perceived stress (Table 2). Participants with clinical signs of AB mostly reported the stress levels “very much” and “extensively”, unlike the others, who reported especially the stress levels “none” and “slightly”.

An increase in stress levels after the beginning of the COVID-19 pandemic was identified in 183 participants (55.79% from the entire study group), divided as follows: 88 participants (representing 44.26%) with self-evaluated bruxism (51 females, 30 males) and 102 participants (representing 55.74%) without self-evaluated bruxism (68 females, 34 males). In summary, 68.22% of participants with self-evaluated bruxism experienced increased stress during this period, compared to only 51.26% of participants without self-evaluated bruxism. A Chi-square test for association was conducted between the presence of self-evaluated bruxism and the report of an increased stress level during the pandemic. The expected cell frequencies were all greater than 5. There was a statistically significant association between self-evaluated bruxism and increased stress, χ^2^(1) = 4.222, *p* = 0.04. Additionally, there were statistically significant correlations between the increased stress status and bruxism episode frequency, U = 15429, z = 2.650, *p* = 0.008, as participants who felt more stressed during the pandemic also reported bruxism episodes with increased frequency. There was no statistically significant difference between increased perceived stress and participants’ gender or age group (*p* > 0.05).

**Figure 4:**
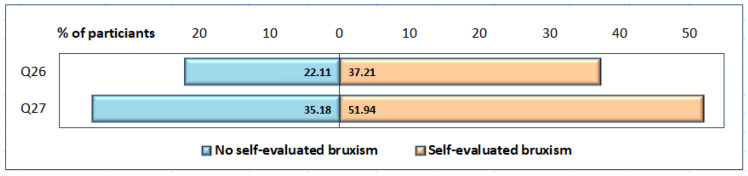
Distribution of participants (expressed as percentage) presenting clinical signs of anxiety: panic [Q26], restlessness [Q27].

Participants’ anxiety was evaluated based on the presence or absence of panic and restlessness sensations (Figure 4). There was a weak association between the presence of self-evaluated bruxism and the sensation of panic (φ = −0.107) and restlessness (φ = 0.033); both associations were not statistically significant (χ^2^(1) = 3.754, *p* = 0.053 for panic; χ^2^(1) = 3.356, *p* = 0.551 for restlessness). There was no statistically significant difference between anxiety presence and participants’ gender or age group (*p* > 0.05).

There were also statistically significant differences between the frequency of bruxism episodes and the level of panic sensation, *p* < 0.0005 (Table 2). Subsequent pairwise comparisons were performed based on Dunn’s procedure with a Bonferroni correction for multiple comparisons. This post hoc analysis revealed significant differences in the frequency of bruxism episodes (*p* < 0.0005) for participants who declared the perceived panic level as “sometimes” (predominant frequency “sometimes”), compared to those who declared the perceived panic levels “rarely” and “never” (both with predominant frequencies “rarely” and “never”). A similar analysis was performed for reported feelings of restlessness, as there were statistically significant differences relative to the frequency of bruxism episodes, *p* < 0.0005 (Table 2). Pairwise comparisons reflected significant differences between the same groups “often” and “rarely”/“never” (*p* < 0.05).

The proportion of participants presenting signs of AB and panic was almost double compared to participants without signs of AB, and the differences were statistically significant; on the other hand, proportions were similar for SB (Table 2). There were also statistically significant differences between the presence of panic and restlessness and the presence of clinical signs of AB, but not the presence of clinical signs of SB (Table 2).

## 4. Discussion

This study addressed a topic of great interest to researchers in the field of dentistry and beyond, given the high prevalence of possible bruxism and the fact that the mechanisms involved in the production of bruxism are multifactorial and not fully known [18,27,28,29,30,31].

The present study was conducted based on a questionnaire, by self-report according to the consensus established in 2012 [23] for the diagnosis and evaluation of bruxism. In this consensus, it was reaffirmed that the gold standard for the diagnosis of SB is represented by polysomnographic records, and the results of examinations with usual diagnostic tools are interpretable; therefore, specialists in the field have proposed a new definition and a graded diagnostic system for SB or AB, represented by “possible”, “probable” and “definite” status [23]. This graded diagnostic system has been proposed for clinical and research purposes. Specialists who have proposed this gradual system of diagnosis considered that “possible” SB or AB is based on self-assessment, on answers to the questions in a questionnaire and/or on anamnesis. “Probable” AB or SB should be diagnosed by self-assessment supplemented by clinical examination. The diagnosis of “definite” or “certain” bruxism is based on self-assessment supplemented by clinical examination and polysomnographic recordings, preferably associated with an audio-video recording for SB, as well as self-assessment, clinical examination, and EMG recordings for AB. It is, therefore, understood that the prevalence of bruxism should include the highest value for “possible” bruxism (self-reported) and the lowest prevalence for “definite” bruxism.

Starting from this consensus in 2012 [23], in March 2017, another international consensus meeting was held to clarify issues related to the classification of bruxism according to the circadian rhythm. This new meeting, which took place in San Francisco in the IADR General Session, was attended by experts in bruxism from around the world [24]. The objectives of this meeting were: to clarify the definition of bruxism, to develop separate definitions for AB and SB, to classify bruxism as a disorder or as a risk factor for other conditions, to re-evaluate the diagnostic system, to establish the reliability, sensitivity, and specificity of each source of information, and to set a new research agenda.

Regarding the definition adopted in 2012, the terms used left room for questions. Thus, while the terms “clenching” and “grinding” of teeth were frequently used by dental practitioners and researchers, the other two terms used, namely “bracing” and “thrusting” the mandible, needed clarification. Lobbezoo et al. [24] quoted Dorland’s medical dictionary [32], noting that “bracing” could mean “holding the maxillary and mandible together or in place” or “doing something stiff, motionless”, while “thrusting” of the mandible was described as “a sudden forced movement”. Translating these terms into the pathophysiology of the MS, “bracing” meant holding the mandible in a certain position, and “thrusting” referred to the forceful movement of the mandible anteriorly or laterally, without dento-dental contacts. The addition of the terms “bracing” and “thrusting” to the previously existing terms of clenching and grinding was in line with the new guidelines stating that bruxism is established mainly centrally, not peripherally, and, therefore, is not determined by peripheral factors such as dental occlusion or temporo-mandibular joint, and involves more than dento-dental contacts [33]. It should also be noted that current examination methods cannot differentiate the activity of the masticatory muscles during clenching from that during grinding, nor during bracing of the mandible from that during thrusting, as these are distinct muscular manifestations. New approaches are needed to better understand the physiology and pathophysiology of such activities of the jaw.

The second aspect discussed at the 2017 meeting concerned the classification of bruxism according to circadian rhythm: SB (night) and AB (daytime) and the formulation of two separate definitions. It is noteworthy that both definitions refer to the activity of the masticatory muscles, thus emphasizing that the contraction disorders of these muscles are at the origin of bruxism, whether it is during sleep or in the awake state. Although the focus is on the activity of the masticatory muscles, in both forms of bruxism, other manifestations may occur, such as changes in heart rate, respiratory parameters and brain activity, but it is recommended that studies on SB or AB focus on the activity of the masticatory muscles.

Both definitions ended with the wording “in physically healthy individuals”, which showed that in most people, bruxism is not a condition (disorder) but a sign of another condition: for example, sleep disorder with rapid movements of the eyes, obstructive sleep apnea, epilepsy, etc., a situation in which the underlying condition requires the attention of a specialist [18]. In addition, although this process is not fully understood, certain manifestations related to bruxism may also have a beneficial effect on the organism, such as in the case of sleep apnea, when bruxism generates the clearing of the upper airways and breathing resumption [34,35], or in stress-induced reactions, as bruxism seems to be able to decrease stress-induced allostatic overload [36].

The correct, relevant assessment of bruxism in an individual is meant, in addition to highlighting the presence or absence of masticatory muscle activity, to determine whether this masticatory muscle activity could become a risk (or a protective) factor for a disorder of the oral health condition. The assessment of bruxism can be non-instrumental or instrumental [24]. Non-instrumental assessment of bruxism is based on self-reporting (questionnaire or anamnesis) and clinical inspection, for both forms of bruxism [37]. Self-reported assessments of AB and SB continue to be the main tool in research and clinical practice. Although there was poor agreement with instrumental assessment especially for SB [38], self-reporting was useful for some applications [24]. Thus, based on self-reporting, it has been established that bruxism may be associated with stress and anxiety (both measured with validated methods) [39], as well as with muscle and joint pain [40,41,42].

Lobbezoo et al. in 2018 [24] considered that there is a limitation on the self-reporting of stress associated with bruxism, as patients might report stress rather than the actual activity of the masticatory muscles. Self-reporting methods should, therefore, be improved to increase the accuracy and reliability of these methods compared to instrument-based methods. Self-reporting can assess the possible presence of AB or SB and the frequency of episodes of bruxism over time. Data on the intensity and duration of bruxism episodes cannot be accurately collected by self-reporting [43].

The assessment of bruxism in the waking state begins by making the patient aware of the significance of teeth grinding and clenching of the jaws. Grinding of teeth can be easily defined as the contact between the teeth of the two arches outside the masticatory and swallowing function, and clenching refers to increased levels of masticatory muscle activity without tooth contact [24]. After explaining these two notions, patients would then be asked to monitor their daily grinding and clenching activities for a period of 1 or 2 weeks. Data collection can be improved by an instrumental assessment method, the momentary ecological assessment (EMA), which provides multiple reports over an observation period [44] and allows the collection of data on the association between bruxism and other manifestations [45]. A more relevant assessment of the muscular activity, especially in the case of SB, may be accomplished by polysomnography (electromyography—EMG, electroencephalography—EEG, electrooculography—ECG), completed with audio and/or video recordings [24,46]. Castroflorio et al. monitored the cardiac, respiratory and brain activity, showing that there are brief sudden modifications of these parameters before an SB event. Thus, to increase the accuracy of SB detection, patients’ heart rates could be recorded through a portable EMG monitoring device [47,48]. On the other hand, according to Atilgan et al., the presence of bruxism may be considered a sign of cardiovascular diseases [49]. Multiple studies investigated the relationship between the oral signs and cardiovascular disease [50,51,52].

The assessment of SB based on self-reporting is easy to perform because it can be based on the statements of the patient, bed partner, or, in the case of children, based on the statements of parents. Patients are asked to monitor their own behavior and record whether they have noticed (or have been told) that they are grinding their teeth, holding their teeth together, or keeping their jaws clenched while sleeping, preferably using a diary. The bed partner may also be asked to keep a diary to record if she/he hears the partner grinding his/her teeth at night. Several patients and bed partner evaluation reports over 1–2 weeks period can provide patient data that may be useful in research and clinical practice.

The present study, conducted by self-reporting based on a questionnaire, was similar to other studies conducted by Flueraşu et al. in 2022, Soares et al. in 2017, Cavallo et al. in 2017 and Quadri et al. in 2015 [25,53,54,55]. To determine the prevalence of possible bruxism, only higher frequencies of bruxism episodes were taken into account, thus avoiding an over-reporting of bruxism, as sporadic episodes do not have pathological significance [56].

The prevalence of bruxism (in all its forms) recorded in this study was 39.33%, comparable to the results obtained by Flueraşu et al. in 2022, also in Romania [53]. Cavallo et al. in 2017 [25] conducted a study on 278 students in Italy based on a questionnaire and recorded a prevalence for AB of 37.9% and for SB of 31.8%, both without significant gender differences, while Quadri et al. in 2015 [55] reported a prevalence of bruxism in up to 83% of dental students at Jazan University. Soares et al. 2017 [54] conducted a study in Brazil on 253 students, based on completing a questionnaire and highlighting the facets of dental wear. According to this study, the prevalence of bruxism estimated as “probable” was 31.6%. That is a lower value than ours, but as we have shown above, it is normal for the prevalence of “probable” bruxism to be lower than that of “possible” bruxism. For the general population, another study reported a lower prevalence, between 8% and 31.4% [57].

Regarding the type of bruxism according to the circadian rhythm, the study showed an important predominance of AB compared to SB, as in the study of Manfredini et al. [58] compared to the study of Fluerasu et al. [53], which showed the predominance of SB.

Regarding the correlation between sex and the prevalence of bruxism, this study showed a very weak association, not statistically significant, while Cavallo et al. and Hublin et al. found a higher association with females [25,59], and Quadri et al. [55] reported a much higher prevalence in males.

The present study demonstrated the association of bruxism with stress and anxiety. The involvement of psychosocial factors, especially stress and anxiety in the production of bruxism, is also discussed in other studies [25,27,28,53,54,60,61,62]. Chemelo et al. conducted a meta-analysis review and concluded that stressed people have a greater predisposition to bruxism [10]. Another representative study associating bruxism with stress was conducted in 2018 by Kuhn et al. [28], also based on a review of the literature from 2007–2016.

Regarding the association between bruxism and anxiety, Polman et al. in 2019 [63] claimed that it was not clear in the literature, but it seemed that some specific symptoms in the spectrum of anxiety disorders could be associated with probable SB. On the other hand, Levartovsky et al. [64], in a study on dental students, showed that there was an association between emotional distress and AB, especially in boys.

Another recorded aspect was the increase in stress levels in study participants during the Covid-19 pandemic, especially in participants with self-evaluated bruxism. This aspect was also assessed in students in Germany by Voltmer et al. [65]; however, the authors reported no significant differences from previous years in terms of stress levels.

Among the major signs associated with self-reported bruxism, this study addressed fatigue and masticatory muscle pain, orofacial pain, and sleep disturbances. Other studies have associated these manifestations, too, as Soares et al. [54] associated bruxism with masticatory muscle pain, Nieto et al. [27] with myofacial pain and temporo-mandibular joint pain, and Ohayon et al. [66] with masticatory muscle discomfort and sleep disorders, while Lavigne et al. 2003 [7] associated bruxism with masticatory muscle disorders in general. Ultimately, these signs are specific to both bruxism and temporo-mandibular disorders and call into question the relationship between bruxism and temporo-mandibular disorders. Several specialists appreciated that, in addition to signs of bruxism such as dental wear, dental cracks and fractures, and prosthetic restoration fractures, in patients with temporomandibular disorder, there are other signs such as joint pain, muscle contracture, joint noises, joint remodeling and headaches [15,41,67].

Manfredini et al. [41] conducted a study in two specialized clinics, in which they followed the coincidence of the signs of temporo-mandibular disorder and self-reported bruxism. The introduction of modern investigative methods such as nuclear magnetic resonance and polysomnography substantially altered the results of the study so that no correlation could be established.

In contrast, Anastassaki et al. [68] conducted a study on 3194 patients and concluded that all symptoms of temporo-mandibular disorder in adults, except for the crackles, were associated with an awareness of clenching and grinding of teeth, and, therefore, with manifestations of bruxism.

Currently, bruxism represents an important topic for dentists, as well as for specialists in other medical areas. A series of studies have revealed the associations between bruxism and various cardiovascular, renal, or digestive diseases [69,70,71].

The limitations of this study are represented by the method of data collection through a questionnaire, without clinical examination and without other paraclinical investigations, and by the level of knowledge of students from different years of study. Raphael KG et al. in 2016 [72] also stated that there is currently evidence of a weak correspondence between self-reported data, clinical evaluation by direct observation methods and state-of-the-art records by polysomnography.

## 5. Conclusions

This study drew attention to the high prevalence of possible bruxism in students attending the Faculty of Dentistry, University of Medicine and Pharmacy of Craiova, and its association with stress and anxiety, especially for awake bruxism. The presence of possible bruxism has been associated with grinding of teeth, tooth sensitivity and several signs of temporo-mandibular disorder: pain in the masticatory muscles or neck muscles. In the context of the pandemic, there was also an increase in stress levels both in students with and without self-reported bruxism.

Even if bruxism is considered a psychic valve, it is of utmost importance that participants increase their awareness levels regarding bruxism’s consequences and take necessary measures to adjust their attitudes towards stress and anxiety, to reduce them as much as possible.

## Figures and Tables

**Table 1:** Statistical results following group distribution analysis by gender, age, frequency of bruxism episodes, and the presence of SB, AB, and combined bruxism.

			Bruxism	
		AB	SB	Combined
Gender	F	Diff = 0.133, ***p* ^1^ = 0.048**	Diff = 0.006, *p* ^1^ = 0.939	Diff = 0.139, ***p* ^1^ = 0.031**
	M			
Age groups (years old)	≤23	U = 1230, z = 0.656, *p* ^2^ = 0.512	U = 1802, z = 0.120, *p* ^2^ = 0.904	U = 1163, z = 0.840, *p* ^2^ = 0.401
	24–25			
	≥26			
Frequency	sometimes	U = 1119.5, z = −0.104, *p* ^2^ = 0.917	U = 1962.5, z = 1.048,*p* ^2^ = 0.295	U = 1213, z = 1.259, *p* ^2^ = 0.208
	often			
	continuously			

^1^ Test of two proportions (Chi-square test of homogeneity). ^2^ Man–Whitney U test.

**Table 2:** Statistical results following group distribution analysis of presence of AB signs, SB signs, frequency of bruxism episodes, and declared stress and anxiety levels.

	Stress Levels	Anxiety Presence	
		Panic	Restlessness
Presence of AB signs	U = 14385.5, z = 3.230, ***p* ^1^ = 0.001**	Diff = 0.217, ***p* ^2^ < 0.0005**	Diff = 0.233, ***p* ^2^ < 0.0005**
Presence of SB signs	U = 6036.5, z = 0.512, *p* ^1^ = 0.609	Diff = −0.049, *p* ^2^ = 0.559	Diff = −0.063, *p* ^2^ = 0.404
Frequency of bruxism episodes	H(4) = 45.507, ***p* ^3^ < 0.0005**	H(4) = 20.595, ***p* ^3^ < 0.0005**	H(4) = 24.609, ***p* ^3^ < 0.0005**

^1^ Man–Whitney U test. ^2^ Test of two proportions (Chi-square test of homogeneity). ^3^ Kruskal–Wallis H test.

## Data Availability

The authors declare that the data for this research are available from the corresponding authors upon reasonable request.
